# Upper incisor position in the sagittal plane when smiling. A photographic and CBCT study

**DOI:** 10.4317/jced.61308

**Published:** 2024-03-01

**Authors:** Asunción Bedoya-Valiente, José-María Llamas-Carreras, Francisco Pastor, Juan-Carlos Rodríguez, Eduardo Espinar-Escalona, José-María Barrera-Mora

**Affiliations:** 1 Dental orthodontic (DDS). University of Sevilla; 2 Doctoral Degree (PhD). Lecturer, University of Sevilla; 3 Department of Ortodoncia. University of Sevilla; 4 Doctoral Degree (PhD). Titular Professor. University of Sevilla; 5 Doctoral Degree (PhD). Assistant Professor. University of Sevilla

## Abstract

**Background:**

The aim of this study was to analyze the anteroposterior position between the upper incisors (UI) and the soft tissues based on photographs in which the head has been oriented along the Frankfort Horizontal Plane.

**Material and Methods:**

Restrospective case-control study carried out by analizing photographic and CBCT images of 109 patientes. The sample was divided into 4 different groups: 21 normocclusive (N), 29 Class II/1st, 29 Class II/2nd y 30 Class III. All patients were positioned using the Frankfurt plane (FH). From this aligned position of the head, a vertical line was drawn perpendicular to the FH passing through the Soft-Tissue Nasion (LN), and the distance in centimeters from of the UI to this vertical line was measured on both the CBCT and the photo of the patient’s profile.

**Results:**

The UI was located in front of the LN in the groups N, Class II/1st y Class III (0,4, 0,2, 0,1cm respectively) and behind the LN in the group Class II/2nd (0,2cm). There were significant differences between the Class II/2nd and Normocclusive groups and Class II/2nd and Class II/1st (*p*<0.001 y *p*=0.004 respectively).

**Conclusions:**

Orthodontic and/or surgical correction of various malocclusions can be planned based on the position of the UI with respect to the LN established in Normocclusive patients.

** Key words:**Upper incisors, facial profile, CBCT, photograph, Frankfurt plane, Soft-Tissue Nasion.

## Introduction

Patient profile analysis is a key factor in many orthodontic and orthognathic surgery treatment decisions. Much of the orthodontic literature has focused on lateral cephalometric analysis since the classic 1948 article by Downs (1,2).

Different combinations of cephalometric measurements have been grouped into a number of “cephalometric analyses,” including Downs (1,2), Steiner (3), Tweed (4,5), Jarabak (6), Sassouni (7), Björk (8), Ricketts (9,10), McNamara (11) and Arnett (12,13). This traditional cephalometry uses internal skeletal landmarks to define points, lines, and/or planes, which are then used to quantify the anteroposterior (AP) position of the jaws and incisors. The use of such internal landmarks has been found to be unreliable, however, due to both errors in identification and variability of their positions between individuals (14-19).

Acknowledgement of these limitations has led others to push for the use of external landmarks such as the soft tissues of the nose, lips, and chin to replace or augment cephalometric analysis (12,20-26).

Furthermore, in recent years, orthodontists have also come to rely heavily on aesthetic judgments based on semi-standardized facial photographs, with these photographs forming part of the routine orthodontic records. The common expectation held by many of these orthodontists is that higher facial attractiveness in profile photographs would be closely associated with ideal cephalometric measurements, whereas both higher and lower values for cephalometric measurements are generally correlated with lower facial attractiveness.

There are therefore numerous studies in the literature that use photographs to evaluate the facial profile. They initially analyzed the sagittal and vertical position of the upper and lower lip, as well as the soft chin (27-35). Of these studies, Stoner (28) described a method for evaluating facial imbalances and establishing criteria for determining the degree of change in facial profile following orthodontic treatment, using a plane that passes through the Soft-Tissue Nasion and Point C (chin).

With the subsequent advent of technical improvements in orthodontics and surgery, the focus has shifted to a greater analysis of the ideal position of the upper incisors as the starting point in a treatment plan. Treatment mechanics can then be planned around an ideal incisor position, with the rest of the teeth being planned around that ideal position. For this purpose, the upper incisors are analyzed from both a frontal and lateral perspective. There is ample literature and consensus regarding the vertical position of the upper incisors in relation to the upper lip at rest and when smiling, and regarding the sagittal position of the upper incisors in relation to bony landmarks (1,9,11,34-40).

However, very few studies have analyzed the anteroposterior position of the upper incisors and the soft tissues.

In one of these studies, studied lateral radiographs to analyze the anteroposterior position of the upper incisor relative to a line that he called the True Vertical Line (TVL), which passes through the Subnasal. Hernández-Alfaro (39), and later Singh (40), analyzed upper incisor position in profile photographs in relation to a line perpendicular to the ground passing through the Soft Tissue Nasion. This handful of studies analyze radiographs and photographs oriented in the natural head position (NHP)12 (36-40), which is the usual head position when looking at an object at eye level on the horizon. However, none of the studies analyze photographs using the Frankfort Horizontal (FH) reference plane. It is therefore worthwhile to carry out a study analyzing the anteroposterior position between the upper incisors and the soft tissues based on photographs in which the head has been oriented along the Frankfort Horizontal Plane.

## Material and Methods

The present study was carried out by randomly selecting patients belonging to an orthodontic clinic.

Four groups of patients were selected: 1) control group of 21 Normocclusive patients (20 females and 1 male); 2) 29 patients with Class II Division 1 malocclusion (20 females and 9 males); 3) 29 patients with Class II Division 2 malocclusion (23 females and 6 males); and 4) 30 patients with Class III malocclusion (12 females and 18 males).

All of the selected patients had photographs of their smile in profile and CBCT radiographs recorded prior to their orthodontic treatment, except for the control group, which underwent CBCT for other non-orthodontic reasons.

The following were the inclusion criteria for each of the groups:

Normocclusive Group: Class I molar, upper osseo-dental discrepancy of 4 mm or less, protrusion of 2 mm or less, no history of prior orthodontic treatment.

Class II Division 1, Class II Division 2, and Class III groups: present a Class II or Class III molar and canine relationship, depending on the group to which they belong.

Patients who presented with previous trauma or restoration of the upper incisor and who did not me*et al*l the established inclusion criteria were excluded.

All CBCT scans were taken on a Kodak 9500 machine using S3D imaging software, with the patients in maximum intercuspidation.

CBCT measurements were analyzed using the Invivo software.

**Figure 1:**
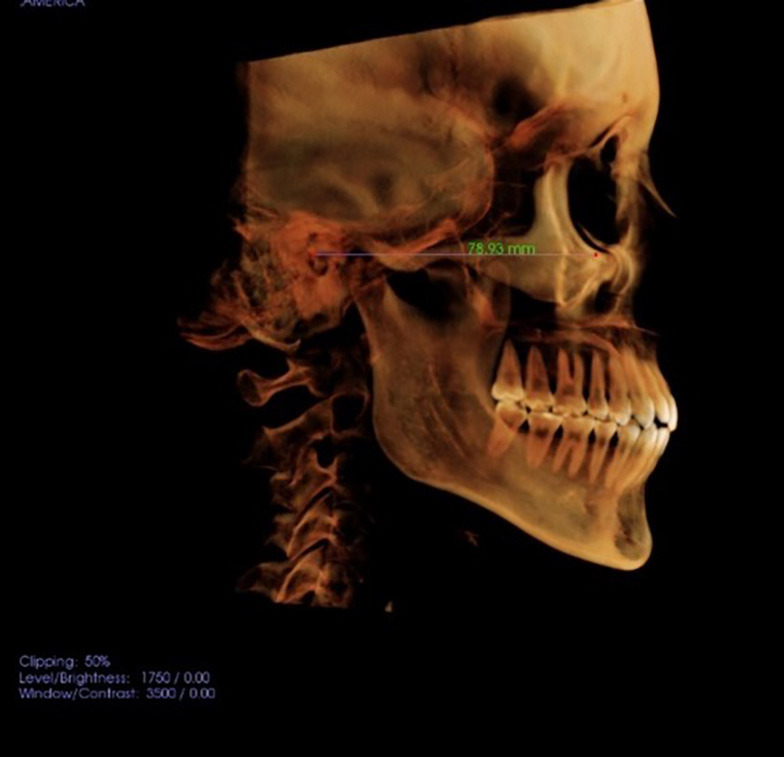
CBCT image of FH-based head alignment.

All CBCT scans were analyzed to reorient the head position in a reproducible manner, using the Frankfurt Horizontal plane (FH) parallel to the ground as a reference; which was traced from the Porion to Orbital points. See Figure 1.

**Figure 2:**
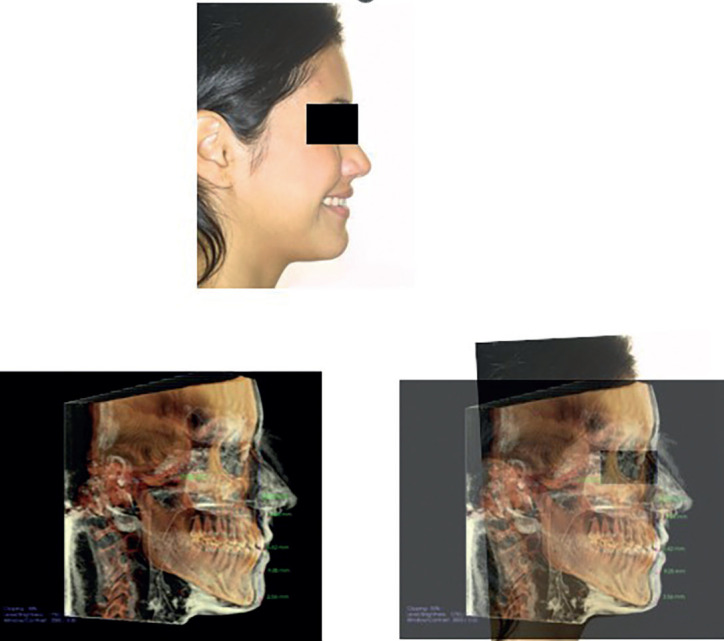
Photographic head realignment based on FH–aligned CBCT scan.

Once the head had been oriented on the CBCT, the photograph of the head was then aligned to replicate the same position based on radiographic and soft-tissue anatomic landmarks. This dynamic alignment process of the face involves aligning the tragus in the 2D facial image with the auditory meatus in the 3D skull image, as described by Austin-Smith and Maples (41), as well as aligning it between the ridge of the nasal bone and the bridge of the nose or between the nasal column and the tip of the nose (42), (Fig. 2).

From this aligned position of the head, a vertical line was drawn perpendicular to the FH passing through the Soft-Tissue Nasion (LN), and the distance in centimeters from the center of the crown of the upper incisor to this vertical line was measured on both the CBCT radiograph (PIR) and the photo (PIF) of the patient’s profile.

**Figure 3:**
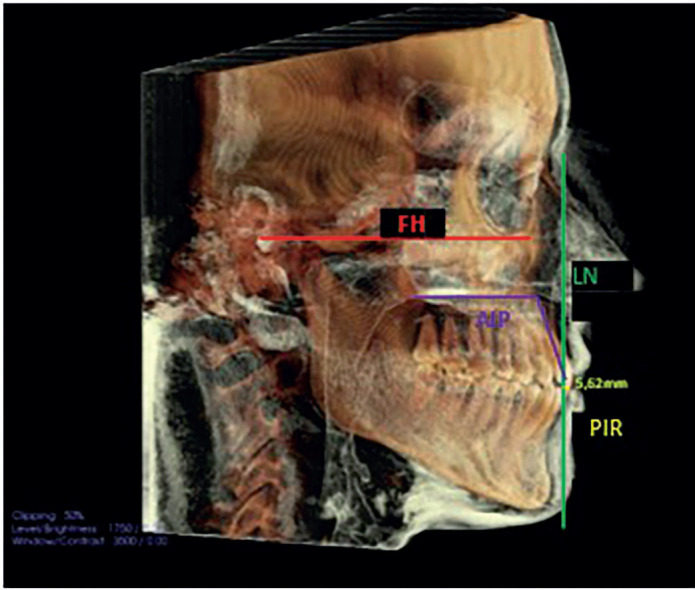
PIR and AIP measurements.

**Figure 4:**
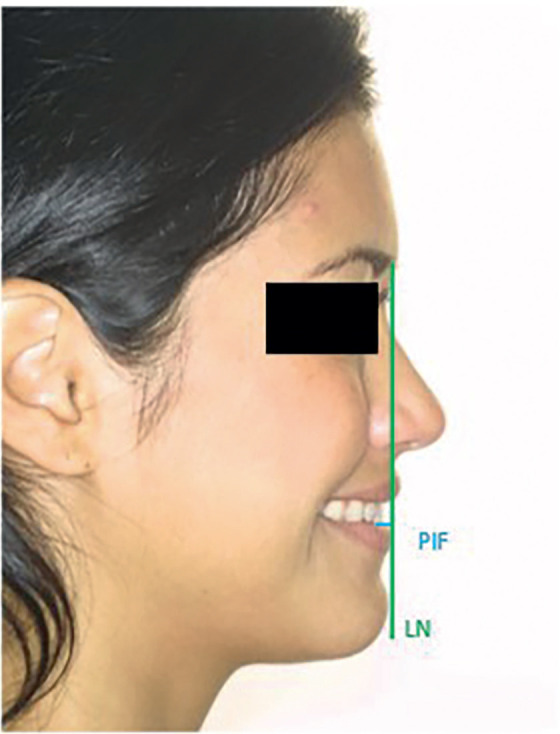
PIF measurement.

**Figure 5:**
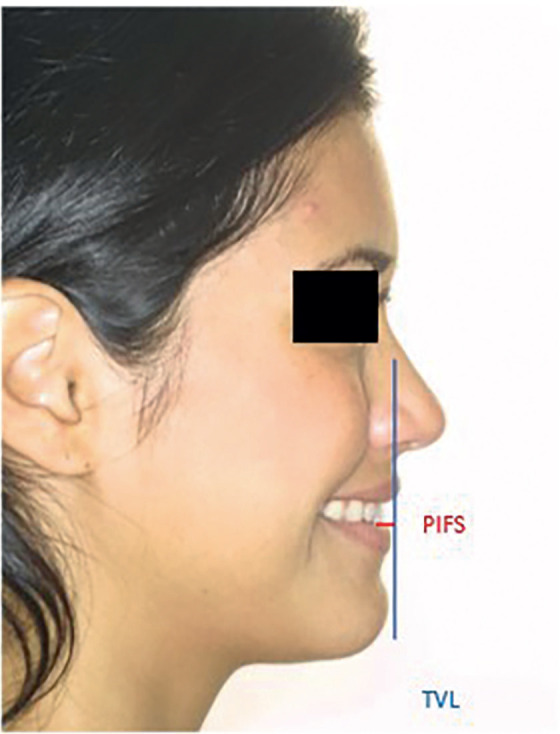
PIFS measurement.

Figures 3, 4, and 5 illustrate the measurements taken during this study.

Values measured in front of this line were established as positive values and those behind the line as negative values.

Measurements were also taken using the CBCT scan to determine whether there were significant differences between the two measurements, which would indicate possible errors in the alignment of the head in the photograph.

The photograph was also used to measure the distance from the upper incisor to the vertical TVL line described by Arnett (PIFS), which also passes through the Subnasale point but without first aligning the head based on the FH base; this was done in order to compare the results of both studies.

Finally, the angulation of the upper incisor with respect to the palatal plane was measured on the CBCT scan (AIP).

-Statistical Analysis

The R statistical software (R Development Core Team, version 3.6.3) was used for statistical analysis of the study data.

A descriptive analysis was conducted, providing distributions of the relative and absolute frequencies for qualitative variables, and measures of position and dispersion for quantitative variables.

Pearson’s chi-squared test was used to determine whether the distribution of the group was the same for each sex. Student’s t-test for independent samples was used to analyze the differences between the sexes in the studied variables.

The four groups were compared using the ANOVA test or the Kruskal-Wallis test, depending on whether or not the normality and homoscedasticity hypotheses were verified.

Similarly, the differences between the measurements for each of the four groups were analyzed using Student’s t-test or the Wilcoxon test for related samples, depending on whether or not the normality hypothesis was verified.

The significance level used was 0.05.

## Results

Table 1 displays the averages of the different measurements obtained for each group.

**Table 1:**
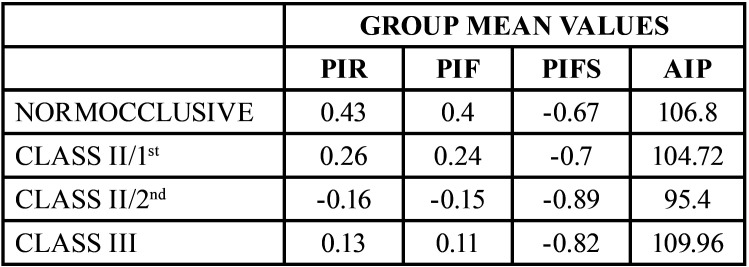
Mean values obtained for each group.

In the Normocclusive group, the upper incisor (UI) was located in front of the Soft-Tissue Nasion (LN) in the PIR and PIF in 90.28% of the individuals, with a maximum distance of 1.02 cm, and behind the LN in 9.52% of patients, with a maximum distance of -0.28 cm.

In the Class II Division 1 group, the UI was located in front of the LN in both the PIR and PIF in 62.07% of patients, with a maximum distance of 1.07 cm, in line with the LN in 17.24% of patients, and behind the LN in 20.69% of patients, with a maximum distance of -0.54 cm.

In the Class II Division 2 group, the UI was located in front of the LN in the PIR and PIF in 31.04% of patients, with a maximum distance of 0.71 cm, in line with the LN in 17.24% of patients, and behind the LN in 51.72% of patients, with a maximum distance of -1.19 cm.

In the Class III group, the UI was located in front of the LN in the PIR and PIF in 53.33% of patients, with a maximum distance of 0.69 cm, in line with the LN in 23.33% of patients, and behind the LN in 23.33% of patients, with a maximum distance of -1.15 cm.

For the PIFS measurement, the UI was located behind the Subnasal Line at a distance of -0.67 cm in the Normocclusive group, -0.70 cm in the CII/1st group, 0.89 cm in the CII/2nd group, and -0.82 cm in the CIII group.

Table 2 displays the results of the measurements obtained for the inclination of the UI in relation to the palatal plane (AIP).

**Table 2:**
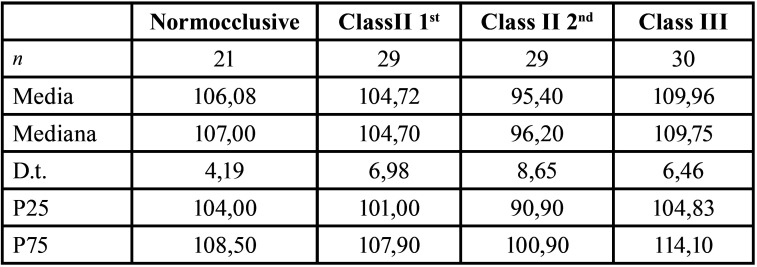
AIP measurement results for each group.

The AIP of the UI was found to be normal in patients in the Normocclusive and Class II/1st groups, retroinclined in the Class II/2nd group, and proinclined in the Class III group. These were the expected results for each group based on the modified Steneir cephalometric analysis.

A comparison of the different measurements taken of each n the different groups yielded the following results.

-Comparison of PIR measurements between the different groups: there were significant differences between the Class II/2nd and Normocclusive groups (*p-value* < 0.001) and Class II/2nd and Class II/1st (*p-value* = 0.004), respectively. On the other hand, there were no significant differences between: Class III and Class II/2nd (*p-value* = 0.079); Class III and Normocclusive (*p-value* = 0.09); Class II/1st and Normocclusive (*p-value* = 0.549); and Class III and Class II/1st (*p-value* = 0.674), respectively (Table 3, Graph 1).

**Table 3:**
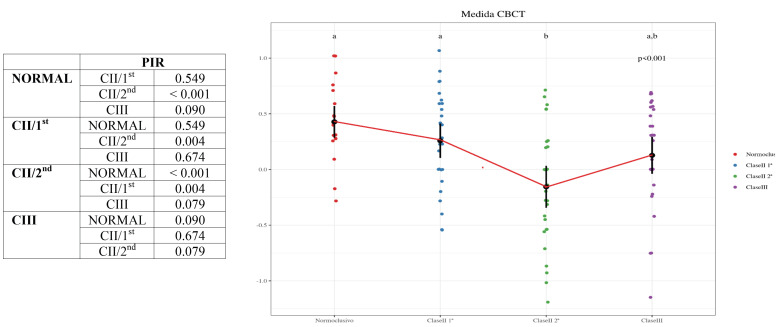
Graph 1. Comparison of PIR measurements between the different groups.

-Comparison of PIF measurements between the different groups: there were significant differences between the Class II/2nd and Normocclusive (*p-value* < 0.001) and Class II/2nd and Class II/1st (*p-value* = 0.004), respectively. On the other hand, there were no significant differences between: Class III and Normocclusive (*p-value* = 0.094); Class III and Class II/2nd (*p-value* = 0.096); Class II/1st and Normocclusive (*p-value* = 0.579); and Class III and Class II/1st (*p-value* = 0.654), respectively (Table 4, Graph 2).

**Table 4:**
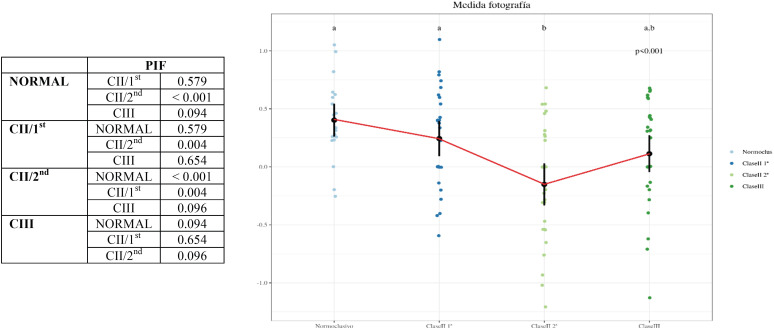
Graph 2. Comparison of PIF measurements between the different groups.

-Comparison of PIFS measurements between the different groups: there were significant differences between Class II/2nd and Class II/1st (*p-value* = 0.036), respectively. On the other hand, there were no significant differences between: Class II/2nd and Normocclusive (*p* value = 0.089); Class III and Class II/1st (*p-value* = 0.286); Class III and Normocclusive (*p-value* = 0.399); Class III and Class II/2nd (*p-value* = 0.668); and Class II/1st and Normocclusive (*p-value* = 0.884), respectively (Table 5, Graph 3).

**Table 5:**
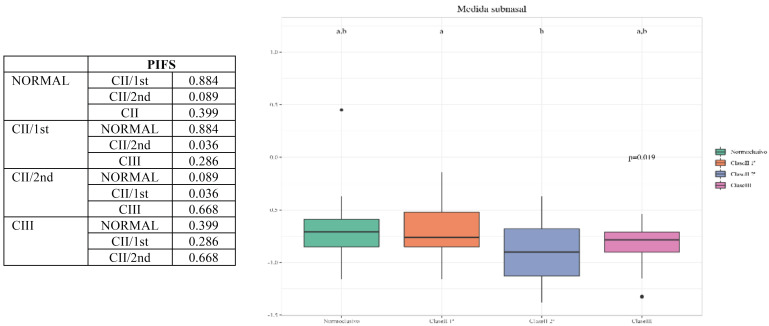
Graph 3. Comparison of PIFS measurements between the different groups.

-Comparison of AIP measurements between the different groups: there were significant differences between Class III and Class II/2nd (*p-value* < 0.001); Class II/2nd and Normocclusive (*p-value* < 0.001); Class II/2nd and Class II/1st (*p-value* < 0.001); and Class III and Class II/1st (*p-value* = 0.036), respectively. On the other hand, there were no significant differences between the following groups: Class III and Normocclusive (*p-value* = 0.237) and Class II/1st and Normocclusive (*p-value* = 0.463), respectively (Table 6, Graph 4).

**Table 6:**
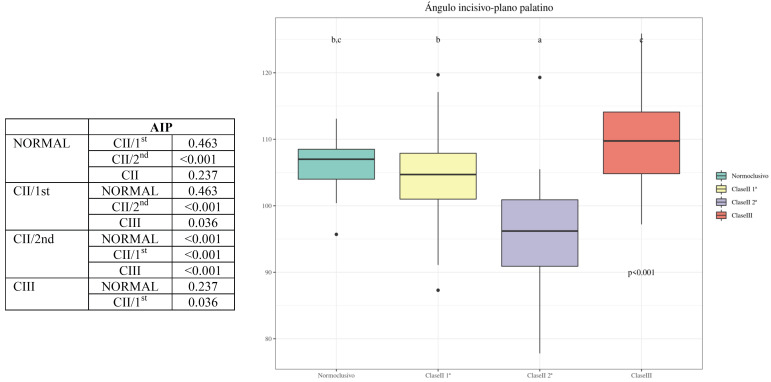
Graph 4. Comparison of AIP measurements between the different groups.

A comparison of the results obtained for the PIR and PIF measurements for each of the groups (Table 7) shows no significant differences between the two measurements in any of the groups, which would indicate that there were no errors in the alignment of the head in the photograph relative to the CBCT.

**Table 7:**
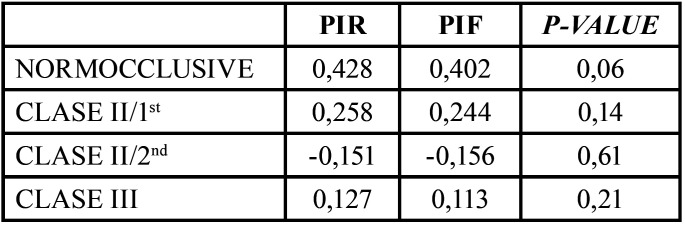
Comparison of PIR and PIF for each group.

Table 8 displays the means of the measurements for each sex within each group and the comparison between them. The Normocclusive group is excluded, as there were not enough cases to compare (20 women vs. 1 man).

**Table 8:**
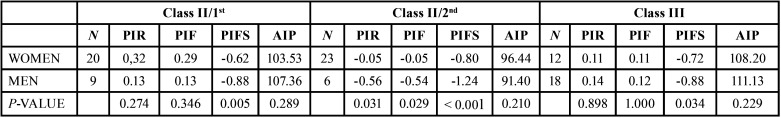
Means for each sex within each group (except Normocclusive group).

The UI is found in front of or in line with the LN in the PIR and PIF in the majority of cases in the Class II/1st (79.31%) and Class III (76.66%) groups, and without significant differences from the Normocclusive group. Furthermore, and also the inclination of the UI in these groups does not differ significantly from that of the Normocclusive group. It can therefore be deduced that the UI is in a correct position with respect to the LN plane in these individuals and its position is not affected by its inclination, which could indicate that the malocclusion in both groups is not due to an abnormal position of the UI but rather of the lower incisor (LI).

Only 5.5% of Class II/1st group individuals who present with the UI in front of the LN, have the UI in a more advanced position than those in the Normocclusive group; this could indicate that the Class II/1st malocclusion is due to an incorrect position of the UI.

Within the 20.69% of individuals belonging to the Class II/1st group who present with the UI behind the LN, in 10.35% of cases the UI is in a more posterior position than those of the Normocclusive group; therefore, in these cases both the UI and the LI present in a more posterior position.

With regard to Class III patients who present with UI behind the LN (23.33%), the UI is more posterior compared to the Normocclusive group (57.14%). Therefore, Class III in these cases could be due only to a more posterior position of the UI or to a more posterior position of the UI in combination with a more anterior position of the LI.

Only in the Class II/2nd group is the UI behind the LN in the PIR and PIF in the majority of cases (51.72%) and with significant differences compared to the Normocclusive group. Of this 51.72%, 73.33% present with the UI in an even more posterior position than patients in the Normocclusive group. In addition, the inclination of the UI shows significant differences with respect to the Normocclusive group; it can therefore be deduced that the position of the UI in the Class II/2nd group is affected by its inclination, in this case retroinclination, and that the UI is not in a posterior position, but only retroinclined.

The remaining 31.04% of individuals in the Class II/2nd group present with the UI in front of the LN, and of these, 55.55% present with the UI even further forward than those in the Normocclusive group. Therefore, Class II/2nd malocclusion in these individuals is due to an abnormal position of the UI.

With regard to the results obtained within each group in relation to sex, in the PIR and PIF measures, significant differences only appear between individuals in the Class II/2nd groups. This is in contrast to the results obtained for the PIFS measurement, in which significant differences appear between individuals in the Class II/1st, Class II/2nd, and Class III groups, which could indicate a greater impact of sex on the measurement of the variability of the subnasal structure.

## Discussion

Our study analyzed the position of the upper incisor with the head oriented based on the Frankfurt Horizontal plane (FH), and not on the Natural Head Position (NHP) as described in the studies carried out by Hernández-Alfaro (39) and Singh *et al*. (40) Lundström *et al*. (43) state that there are variations in NHP recordings due to the difficulty subjects have in reproducing a natural mean head position, as do Moorrees *et al*. (44) and Downs *et al*. (45).

Likewise, other authors such as Halazonetis *et al*. (56) in their study state that NHP is influenced by changes in chin position; Dohyun Cho *et al*. (57) state that changes in NHP appear in Class III patients before and after correction by orthognathic surgery; and Kumar *et al*. (58) describe changes to the natural cranial posture of the subjects in the study depending on the malocclusion they present. Arnett (55) and McLaughlin (59) also recommend recommended adjusting head position during NHP imaging because patients with Class II and Class III facial types tend to compensate for their head position. For example, an individual with Class II mandibular retrognathism may habitually tilt the head backward to mask the Class II appearance. The clinician must identify these individuals and adjust their head position for record-taking to provide a more reliable basis for cephalometric analysis of these individuals. Therefore, when NHP is uncertain, FH should be used to correct this position, as head positioning is important for the correct measurement of distances and angles in lateral cephalometry, both for lateral cephalometries obtained by conventional radiography in 2D and for those obtained using Cone Beam Computed Tomography (CBCT) in 3D (46-53). A few authors have developed techniques in their studies to replicate NHP in patients (62-64); Xia *et al*. (62,63) used a 3D laser scanner to record the surface geometry and absolute positioning of the facial soft tissues while the patient maintained the NHP and then aligned these models based on these surface findings (61). Although recording the NHP using a laser scanner was very accurate, this method is impractical for routine clinical practices because it is very bulky and expensive (62). In their study, Schatz *et al*. (64) used a guiding sensor attached to the patient’s teeth through a bite block to reproduce the NHP; although the guiding sensor method is inexpensive relative to the laser scanning method, it requires the construction of a bite block, and this severely alters the states of the upper and lower lips while capturing the CBCT image. Other authors such as Tiam *et al*. (65) and Weber *et al*. (66) reliably replicated NHP by conforming to a self-balanced head position when acquiring photographic and 3D images of the study subjects. But the present study used previously acquired photographic and 3D images, so it was not possible to adjust NHP in the subjects under study.

Arnett *et al*. (13,14) reported using planes passing through the subnasal to analyze the position of the UI. But this plane can be affected in cases of maxillary hypoplasia, as can other planes based on soft tis-sue structures such as the nose, lips, and chin, which may not reflect the correct position of the upper incisor due to the variability in their thicknesses and lengths. In this sense, Ayala *et al*. (38), in their study of cases with lack of development of the middle third characterized by a depression of the cheek and a retrusion of the upper lip, in which the subnasal point will also be retruded, determined an ideal sub-nasal point from which a line can be drawn us to reliably evaluate the sagittal position of the lips and the soft chin (38). Therefore, for the purposes of the present study, like Hernández-Alfaro (39) and Singh (40), a plane passing through the Soft-Tissue Nasion is used, as it is not subject to this variability, nor is it affected in cases of maxillary hypoplasia.

In the present study, in the Normocclusive group, the UI appear in front of the LN in the PIR and PIF measures in 90.28% of patients. This is similar to the results obtained in the studies by Hernández-Alfaro (39) and Singh (40), although their patients were not classified within the Normocclusive group. In Singh’s study (40), the UI were ahead of LN in 67% of the patients in the study group, which consisted of individuals who needed orthodontic treatment without being classified in terms of the malocclusions they presented, so the results of their study group cannot be compared with those of the present study group.

Zhou *et al*. (58) also analyzed the position of the UI with respect to a vertical line passing through the NA and perpendicular to the FH in a group of Chinese women with aesthetic profiles after undergoing orthodontic treatment. Within their study, in the group classified as Class I and Class II/1st Angle, they observed that the UI were in front of this vertical line. This is similar to the results obtained in the present study, where the majority of individuals belonging to the Normocclusive group (classified as Class I) and the Class II/1st group have the UI ahead of the LN (90.28% and 62.07%, respectively).

Arnett *et al*. (14) studied the position of the UI in 46 individuals classified as Class I (20 men and 26 women) with respect to the TVL (line passing through the subnasal) and found that the UI fell between 7-11 mm behind the TVL in men and between 10-14 mm behind the TVL in women. The results of the present study showed that in the PIFS measurement the UI was found to be 8.8 mm behind the TVL in the Normocclusive group in men and 6.3 mm behind the TVL in women. However, the two studies cannot be compared because there was only one male patient in the Normocclusive group in the present study.

When analyzing the results obtained in the present study with respect to the Subnasal plane (PIFS) between the different groups, the same significant differences between groups do not appear as those obtained with respect to the Soft-Tissue Nasion plane (PIR and PIF), since there are no significant differences in the PIFS measurement between the Normocclusive group and any of the other groups. The only significant differences were between the Class II/1st and Class II/2nd groups. These differences may be due to the fact that in order to analyze the PIFS measurement, the head was not realigned based on FH, but rather the measurements were taken in NHP, and there may have been modifications made to the NHP by the Class II/1st and Class II/2nd patients to mask their respective malocclusions, in addition to the fact that the subnasal plane may be affected in cases of maxillary hypoplasia.

## Conclusions

– UI position is a key factor in both orthodontic and orthognathic surgery planning.

– The position of the SI relative to the LN in individuals from the Normocclusive group is the same as that obtained by other authors, so the LN could be a landmark for analyzing the correct position of the UI.

– Orthodontic and/or surgical correction of various malocclusions can be planned based on the position of the UI with respect to the LN established in Normocclusive patients.

– When the UI are both in the correct position and inclination with respect to the Normocclusive group, the malocclusion would not be due to an incorrect position of the UI but of the lower incisor (LI), and it is the latter that should be corrected in treatment.

– When the UI are in a more anterior or posterior position than those in the Normocclusive group, and/or the inclination is not correct, at least the UI should be corrected in treatment.

– The present study does not include enough male individuals in the Normocclusive group, so a future study with a larger number of male Normocclusive individuals may be necessary.
